# Association between Blood Mercury Concentration and Prevalence of Borderline Hypercholesterolemia among Adolescents: The Korea National Health and Nutrition Examination Survey (KNHANES) 2010–2013 and 2016

**DOI:** 10.3390/toxics9100242

**Published:** 2021-09-29

**Authors:** Taiyue Jin, Eun Young Park, Byungmi Kim, Jin-Kyoung Oh

**Affiliations:** 1 Division of Cancer Prevention, National Cancer Control Institute, National Cancer Center, Goyang-si 10408, Gyeonggi-do, Korea; taewol@ncc.re.kr (T.J.); kbm5369@ncc.re.kr (B.K.); jkoh@ncc.re.kr (J.-K.O.); 2 Department of Cancer Control and Population Health, Graduate School of Cancer Science and Policy, National Cancer Center, Goyang-si 10408, Gyeonggi-do, Korea

**Keywords:** mercury (Hg), dyslipidemia, hypercholesterolemia, hyper-LDL cholesterolemia, adolescents

## Abstract

There is limited evidence on the association between blood mercury (Hg) concentration and the risk of borderline dyslipidemia in adolescents. Here, we investigated the association between blood Hg concentration and the prevalence of borderline dyslipidemia among Korean adolescents. A total of 1559 participants (806 boys and 753 girls) aged 10–18 years who cross-sectionally enrolled in the Korea National Health and Nutrition Examination Survey (KNHANES) 2010–2013 and 2016 were included in this study. Hg concentrations (µg/L) in whole blood samples were measured. The geometric mean (GM) of the blood Hg concentration was 1.88 µg/L. It showed a 63% higher prevalence of borderline hypercholesterolemia (total cholesterol (TC) 170–199 mg/dL) per unit of natural log-transformed blood Hg concentration in boys (95% CI = 1.10–2.41), but not in girls. When a categorical model was applied, the positive association with the prevalence of borderline hypercholesterolemia was also persistant in boys (OR (95% CI) for 2nd and 3rd tertiles (Hg concentration 1.532–11.761 µg/L) vs. 1st tertile (Hg concentration 0.192–1.531 µg/L): 1.92 (1.19–3.10)), but not in girls. This finding suggests that blood Hg concentration might result in a higher prevalence of borderline hypercholesterolemia among adolescents and more stringent public health actions should be taken for the reduction of Hg exposure to prevent dyslipidemia from early-childhood, despite the need of further study to evaluate a causal relationship between blood Hg concentration and the risk of dyslipidemia.

## 1. Introduction

Mercury (Hg) is a heavy metal ubiquitously distributed in nature, i.e., air, ocean, and soil. Environmental exposure to Hg occurs in daily life, such as with the consumption of foods grown in contaminated oceans (e.g., fish, shellfish, etc.), in air pollution, dental amalgams, and in the use of industrial products (e.g., batteries, lamps, etc.). Hg exposure has been a growing concern as it may be related to several adverse effects on health, including neurodegenerative diseases [1], cardiovascular disease (CVD) [2], kidney diseases [3], type 2 diabetes mellitus (T2DM) [4], and all-cause mortality [5,6].

Worldwide, dyslipidemia is one of the established risk factors for CVD, the top cause of death in adults [7]. According to the results from the NCD Risk Factor Collaboration (NCD-RisC) database, plasma total cholesterol (TC) and non-high-density lipoprotein (HDL) cholesterol levels have globally decreased from 1980 to 2018 in both men and women [8,9]. During the past three decades, the age-standardized mean plasma lipid levels reduced by approximately 0.21 mmol/L (8.1 mg/dL) for TC levels, and 0.04 mmol/L (1.5 mg/dL) for non-HDL cholesterol levels in men, and 0.16 mmol/L (6.2 mg/dL) for TC levels and 0.09 mmol/L (3.5 mg/dL) for non-HDL cholesterol levels in women. The decreases in plasma lipid levels mainly occurred in America and Europe, whereas increasing trends of these levels have been observed in the Western Pacific region, especially in East Asia. Particularly, the prevalence of dyslipidemia in Korea has increased from 8.0% in 2005 to 22.3% in 2019 among adults over 30 years of age [10,11].

Previous epidemiologic studies have demonstrated that the development and progression of CVD may begin in early life, although it usually occurs in adults [12,13]. Hence, it is important to screen blood lipid levels from early-childhood, in order to reduce the long-term disease burden. Several studies investigated the association between Hg exposure and the risk of dyslipidemia in adolescents [14,15,16]. A cross-sectional study including 6–19-year-old children and adolescents enrolled in the US National Health and Nutrition Examination Survey (NHANES) 2011–2014 suggested that the prevalence of hypercholesterolemia was significantly higher among individuals in the highest quartile of serum Hg concentration compared with those in the lowest quartile [14]. However, there was no significant association between blood Hg concentration and the prevalence of hypertriglyceridemia or hyper-low-density lipoprotein (LDL) cholesterolemia.

The 2011 US National Heart, Lung, and Blood Institute guidelines for cardiovascular health and risk reduction in children and adolescents suggested that, TC, triglyceride (TG), and LDL-cholesterol levels should be classified as acceptable, borderline high, and high, whereas HDL-cholesterol levels should be grouped into acceptable, borderline low, and low among adolescents [17]. Briefly, borderline dyslipidemia was defined as a borderline high level of TC, TG, or LDL-cholesterol, or borderline low level of HDL-cholesterol. Overt dyslipidemia was ascertained if an adolescent had a high level of TC, TG, or LDL-cholesterol, or a low level of HDL-cholesterol. To our knowledge, there was no epidemiologic study identifying the associations between Hg exposure and risk of borderline dyslipidemia or overt dyslipidemia in adolescents.

Here, we hypothesized that environmental exposure to Hg results in borderline dyslipidemia, as well as overt dyslipidemia in adolescents. Therefore, in this study, we conducted a cross-sectional study to evaluate the association between blood Hg concentration and the prevalence of borderline dyslipidemia among adolescents aged 10–18 years in Korea.

## 2. Materials and Methods

### 2.1. Study Participants

This study is based on the data acquired from the Korea National Health and Nutrition Examination Survey (KNHANES), which is an ongoing national survey to assess health and nutritional status, conducted by the Korea Disease Control and Prevention Agency (KDCA). Details of the KNHANES have been described previously [18].

**Figure 1:**
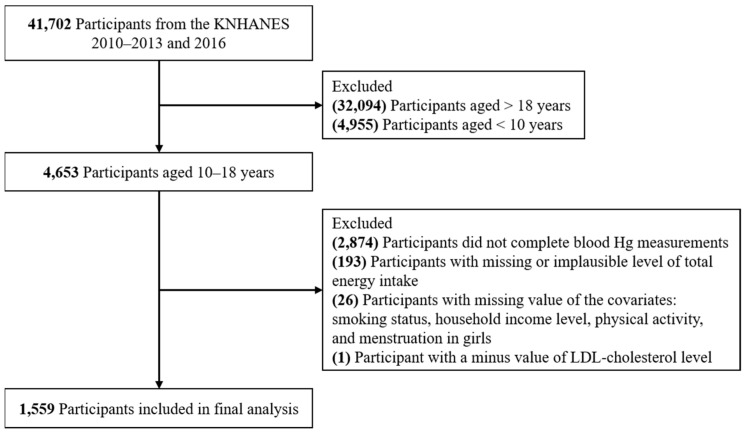
Flow diagram of the study participants.

A total of 41,702 participants (18,926 males and 22,776 females) with blood Hg measured in the KNHANES 2010–2013 and 2016 were initially included. We excluded participants who were adults (*n* = 32,094) or children (*n* = 4955); those who did not complete blood Hg measurements (*n* = 2874); those with missing (*n* = 182) or implausible levels of total energy intake (±3 standard deviations [SDs] from the natural log-transformed mean) (*n* = 11); and those who did not provide information on the covariates, including smoking status (*n* = 12), household income level (*n* = 10), physical activity (*n* = 3) or menstruation in girls (*n* = 1). Furthermore, one participant with a minus value of LDL-cholesterol level was excluded. As a result, a total of 1559 participants (806 boys and 753 girls) aged 10–18 years were included in the final analysis. A flow diagram of the study participants is shown in Figure 1. All the participants have provided informed consent.

The study protocol was approved by the Institutional Review Board (IRB) of the KDCA (2010-02CON-21-C, 2011-02CON-06-C, 2012-01EXP-01-2C, 2013-07CON-03-4C).

### 2.2. Laboratory Analysis

Blood samples were collected after fasting for at least 8 h. The Hg concentration (µg/L) in whole blood samples was measured by the gold-amalgam collection method using DMA-80 (Milestone, Bergamo, Italy). The limit of detection (LOD) for the blood Hg was 0.158 µg/L. There was no participant with an Hg concentration below the LOD. Further information on internal quality assurance and control can be found elsewhere [19]. The Hitachi Automatic Analyzer 7600-210 (Hitachi, Tokyo, Japan) was used to assess serum TC (mg/dL) and TG (mg/dL) levels by enzymatic method and HDL-cholesterol (mg/dL) levels by homogeneous enzymatic colorimetric method.

### 2.3. Ascertainment of Cases

Serum LDL-cholesterol levels were calculated using the Friedewald formula [20]. According to the 2011 US National Heart, Lung, and Blood Institute guideline [17], values of lipid and lipoprotein were categorized into 3 groups: acceptable, borderline high, and high groups for TC, TG, and LDL-cholesterol levels and acceptable, borderline low, and low groups for HDL-cholesterol levels (Table S1). For TC, TG, and LDL-cholesterol levels, the borderline high group was defined as follows: TC 170–199 mg/dL (4.3–5.1 mmol/L); TG 90–129 mg/dL (1.0–1.5 mmol/L); LDL-cholesterol 110–129 mg/dL (2.8–3.3 mmol/L). Meanwhile, the high group was ascertained with values of lipid and lipoprotein as follows: TC ≥ 200 mg/dL (5.1 mmol/L); TG ≥ 130 mg/dL (1.4 mmol/L); LDL-cholesterol ≥ 130 mg/dL (3.4 mmol/L). For HDL-cholesterol levels, the borderline low and low groups were defined as 40–45 mg/dL (1.0–1.2 mmol/L) and <40 mg/dL (1.0 mmol/L), respectively. On the other hand, borderline dyslipidemia was defined if participants classified into any one of the borderline high (for TC, TG, and LDL-cholesterol levels) or borderline low (for HDL-cholesterol levels) groups, whereas those who classified into any one of the high (for TC, TG, and LDL-cholesterol levels) or low (for HDL-cholesterol level) groups were defined as overt dyslipidemia. Furthermore, dyslipidemia was defined as borderline dyslipidemia and overt dyslipidemia combined.

### 2.4. Covariates

Body mass index (BMI, kg/m^2^) was calculated as body weight (kg) divided by the square of height (m). Total energy intake (kcal/day) for each participant was estimated using a 24 h dietary recall. Household income level was categorized in quintiles according to sample household income levels in the KNHANES. We calculated the metabolic equivalent (MET)-min/week by multiplying the total minutes spent in each activity per week by the metabolic cost in METs. Participants were grouped into inactive, minimally active, and active groups according to the International Physical Activity Questionnaire (IPAQ) and Global Physical Activity Questionnaire (GPAQ) scoring protocols [21,22]. The KNHANES did not assess smoking status, alcohol consumption or physical activity, etc., for participants aged 10–11 years. Thus, they were categorized into never smokers and physically inactive groups because the prevalence of ever smoking experience was 4.8% among adolescents before the age of 12 in Korea [23], and the large majority of them were physically inactive [24].

### 2.5. Statistical Analysis

Blood concentrations of Hg was natural log-transformed due to the skewed distribution. Socio-demographic, anthropometric, lifestyle, and clinical characteristics were presented as mean ± standard error (SE) and frequency (percentage) for continuous and categorical variables, respectively. Differences in baseline characteristics between without, borderline, and overt dyslipidemia were estimated using ANOVA tests and Rao-Scott Chi-Square tests for continuous and categorical variables, respectively [25]. Additionally, comparisons of characteristics according to sex were performed using independent t-tests for continuous variables and Rao-Scott Chi-Square tests for categorical variables.

The geometric mean (GM) and 95% confidence interval (CI) of blood Hg concentration were calculated using PROC SURVEYMEANS [26].

Odds ratios (ORs) and 95% CIs were calculated using PROC SURVEYLOGISTIC [27]. We examined the linear associations between blood Hg concentration and prevalence of borderline hypercholesterolemia and borderline hyper-LDL cholesterolemia. In addition, we also estimated the associations for hypercholesterolemia and hyper-LDL cholesterolemia or overt hypercholesterolemia and overt hyper-LDL cholesterolemia (Tables S4 and S5). Since there was no significant difference in blood Hg concentration according to hypertriglyceridemia and hypo-HDL cholesterolemia (Table S2), we did not conduct the further analyses. Categorical models were applied to compare the prevalence between participants in the 2nd and 3rd tertiles of blood Hg concentration and those in the 1st tertile. Groups with blood Hg concentrations in the 1st tertile were regarded as the reference group. We performed univariate analysis (model 1) and multivariate analysis (model 2) adjusted for age (year, continuous), sex (for boys and girls combined), BMI (kg/m^2^, continuous), survey year (2010, 2011, 2012, 2013, and 2016), total energy intake (kcal/day, continuous), smoking status (never smokers and smokers), household income level (quintile 1, 2, 3, 4, and 5), physical activity (inactive, minimally active, and active), and menstruation (for girls, premenarcheal and post-menarcheal). All the analyses were adjusted for sampling weights. All statistical analyses were conducted using SAS version 9.4 (SAS Institute Inc., Cary, NC, USA), and a *p*-value < 0.05 in two-sided tests was considered as a significant difference.

## 3. Results

### 3.1. Baseline Characteristics

Of the 1559 participants, 395 (25.3%) and 130 (8.3%) were classified into borderline and overt hypercholesterolemia, respectively. In addition, 224 (14.4%) and 93 (6.0%) adolescents were identified as borderline and overt hyper-LDL cholesterolemia, respectively.

Table 1 shows the baseline characteristics of the study participants according to TC and LDL-cholesterol levels. The mean age and BMI were 14.2 years and 20.9 kg/m^2^, respectively. Participants with overt hypercholesterolemia were more likely to be younger, girls, obese, never smokers, and physically active, and had a higher total energy intake. Additionally, overt hypercholesterolemia was more frequent in the premenarcheal girls. On the other hand, participants with overt hyper-LDL cholesterolemia were more likely to be girls and obese, and had higher household income levels and total energy intake than those with normal range of LDL-cholesterol levels. Blood Hg concentrations in both the participants with overt hypercholesterolemia and overt hyper-LDL cholesterolemia were higher than those with a normal range of TC and LDL-cholesterol levels. Characteristics according to TG and HDL-cholesterol levels were shown in Table S2. There was no significant difference in blood Hg concentration according to hypertriglyceridemia or hypo-HDL cholesterolemia.

### 3.2. Distribution of Blood Hg Concentration and Differences in Baseline Characteristics

The GM of blood Hg concentration was 1.88 µg/L (95% CI = 1.82–1.94) (Table 2). Notably, boys had higher blood Hg concentrations than girls. Differences in covariates according to blood Hg distribution were examined (Table S3). Compared with participants in the lowest tertile of blood Hg, those in the highest tertile were more likely to be obese and had a higher total energy intake. Moreover, there were more postmenarcheal girls in the lowest tertile of blood Hg than in the highest tertile.

### 3.3. Baseline Characteristics According to Sex

Socio-demographic, anthropometric, lifestyle, and clinical characteristics according to sex are shown in Table 3. Compared with girls, boys were more likely to be obese and physically active. Girls had higher TC, LDL-cholesterol, TG, and HDL-cholesterol levels than boys.

### 3.4. Associations between Blood Hg Concentration and Prevalence of Borderline and Overt Hypercholesterolemia

Associations between blood Hg concentration and the prevalence of hypercholesterolemia are presented in Table S4. It showed a significant higher prevalence of hypercholesterolemia in boys (OR (95% CI) per unit of natural log-transformed blood Hg concentration: 1.74 (1.23–2.46)). When separated with borderline and overt hypercholesterolemia, the positive associations persisted in boys (Table 4 and Table S5). With the increment of per unit of natural log-transformed blood Hg concentration, boys had a 63% higher prevalence of borderline hypercholesterolemia (95% CI = 1.10–2.41). A significant association between blood Hg concentration and the prevalence of overt hypercholesterolemia was also observed (OR = 2.04; 95% CI = 1.15–3.64). In categorical models, compared with boys in the 1st tertile of blood Hg concentration, those in the 2nd and 3rd tertiles had a higher prevalence of hypercholesterolemia (OR = 2.02; 95% CI = 1.30–3.14), furthermore, borderline hypercholesterolemia (OR = 1.92; 95% CI = 1.19–3.10), and overt hypercholesterolemia (OR = 2.36; 95% CI = 1.00–5.54). However, no significant association was observed between blood Hg concentration and the prevalence of borderline or overt hypercholesterolemia in girls.

### 3.5. Associations between Blood Hg Concentration and Prevalence of Borderline and Overt Hyper-LDL Cholesterolemia

Blood Hg concentration was also positively associated with the prevalence of hyper-LDL cholesterolemia in boys (OR (95% CI) per unit of natural log-transformed blood Hg concentration = 1.65 (1.06–2.57)) (Table S4). Likewise, boys in the 2nd and 3rd tertiles had an 88% higher prevalence of hyper-LDL cholesterolemia compared with those in the 1st tertile (OR = 1.88; 95% CI = 1.10–3.20). However, there was no significant association between blood Hg concentration and the prevalence of borderline hyper-LDL cholesterolemia, in neither the continuous model nor the categorical model (Table 4). When separated with sex, a positive association between blood Hg concentration and prevalence of overt hyper-LDL cholesterolemia was observed in boys, but not in girls (Table S5). Boys had a 3.20-fold higher prevalence of overt hyper-LDL cholesterolemia per unit of natural log-transformed blood Hg concentration (95% CI = 1.55–6.62). When a categorical model was applied, the OR (95% CI) for 2nd and 3rd tertiles vs. 1st tertile was 2.64 (0.94–7.36).

## 4. Discussion

In this study, we found that blood Hg concentration was associated with the prevalence of borderline hypercholesterolemia in adolescents, and the positive associations were prominent in boys. To the best of our knowledge, the present study is the first evaluation of the associations between environmental exposure to Hg and the prevalence of borderline dyslipidemia in adolescents. These meaningful findings suggest that the reduction of Hg exposure might be important on a public health perspective for the prevention of dyslipidemia in adolescents.

As with adults, the increase in plasma lipid levels among adolescents has been consistently observed in the Western Pacific region, mainly in East Asia [8,9]. In Korea, a significant increase in the prevalence of overt dyslipidemia from 2007 to 2018 was observed in adolescents aged 10–18 years [28]. For example, the prevalence of overt hypercholesterolemia increased from 6.29% to 8.45% in boys, and from 7.80% to 12.43% in girls during the decades. Likewise, the prevalence of overt dyslipidemia increased from 18.8% in 2004 to 28.9% in 2014 among adolescents aged 10–18 years in China [29]. Additionally, in Japanese children and adolescents, a substantial increase in overt hyper-non-HDL cholesterolemia from 2007 to 2017 was observed in both boys (6.1% and 3.1% at age 10 and 13, respectively) and girls (3.8% and 4.6% at age 10 and 13, respectively) [30]. These increases in the prevalence of dyslipidemia enforce the need for public health interventions for lipid management in children and adolescents.

In recent studies, strict management from the pre-stage of chronic disease is being emphasized in disease prevention. For example, a meta-analysis of 17 cohort studies found that individuals with prehypertension (120–139 mmHg for systolic blood pressure (SBP) or 80–89 mmHg for diastolic blood pressure (DBP)) had a 43% higher risk of CVD compared with those with an optimal blood pressure [16]. On the other hand, the 2011 US National Heart, Lung, and Blood Institute guideline pointed out that it is important to screen lipid levels in children and adolescents since even normal to mild elevations in lipid levels are related to the risk of chronic disease, such as obesity and metabolic syndrome [17]. However, to date, evidence for borderline dyslipidemia risk factors was still limited. In this study, we are the first to demonstrate that environmental Hg exposure may result in borderline hypercholesterolemia among adolescents.

Only a few studies reported the association between Hg exposure and the risk of overt hypercholesterolemia in children and adolescents. In line with our results, a Korean study which used data from the KNHANES 2010–2013 and 2016, also observed a similar positive association between whole-blood Hg concentration and the prevalence of overt hypercholesterolemia [31]. Boys, but not girls, aged 10–19 years had a significant higher prevalence of overt hypercholesterolemia (TC ≥ 200 mg/dL) with the increment of blood Hg concentration (OR (95% CI) for 4th quartile vs. 1st quartile: 3.72 (1.03–13.4)). Although the data we used was the same as that in the Korean study, as emphasized above, we paid more attention to the prevalence of borderline hypercholesterolemia (TC 170–199 mg/dL) resulted by environmental Hg exposure in adolescents. Further investigation is needed to clarify the Hg toxicity on the risk of borderline dyslipidemia among children and adolescents.

For the association between Hg exposure and the risk of overt hyper-LDL cholesterolemia in adolescents, there existed inconsistent results between the current and previous studies. Two cross-sectional studies based on the US NHANES data have investigated the association in children and adolescents aged 6–19 years and 12–19 years, respectively [14,15]. Both studies reported a null association between Hg exposure and the prevalence of overt hyper-LDL cholesterolemia, although a strong positive association among boys was found in this study. Findings on the association between environmental Hg exposure and the risk of overt hyper-LDL cholesterolemia need to be replicated in further prospective cohort studies.

The adverse effects of Hg on lipid and lipoprotein levels might be linked to several mechanisms. A well-established hypothesis of Hg toxicity is oxidative stress (OS) induction, which is implicated in CVD [32]. Paraoxonase 1 (PON1), an enzyme exclusively located on HDL-cholesterol, acts as an antioxidant agent [33]. A US cohort study with at least 3 years of follow-up reported that serum PON1 was inversely related to total plasma oxidized fatty acid levels, and was associated with a lower incident risk of major adverse cardiovascular events (MACE), including nonfatal and fatal myocardial infarction, nonfatal and fatal stroke, and all-cause mortality, suggesting an atheroprotective effect of PON1 [34]. Evidence from experimental and epidemiologic studies has suggested that Hg exposure may inactivate PON1 and lead to lipid peroxidation [35,36]. In a cross-sectional study, a total of 896 Inuit adults, who are highly exposed to methyl mercury (MeHg) from a seafood-based diet, were enrolled from Nunavik, Canada [37]. After being adjusted for potential confounders, an inverse association between whole-blood Hg concentration and plasma PON1 activity was observed. Additionally, chronic Hg exposure may also inhibit glutathione peroxidase activity, another antioxidant enzyme, and increase oxidative damage [38]. Another possible mechanism is adipogenesis dysregulation [36]. Peroxisome proliferator-activated receptors (PPARs), including PPARα, PPARδ, and PPARγ, are a subgroup of nuclear hormone receptors which play an important role in lipid metabolism and inflammation [39]. For example, PPARα regulates the expression of genes involved in lipoprotein metabolism enhancing the clearance of lipids through the liver [40]. Hg exposure may affect lipid metabolism through the down-regulation of the mRNA expression of PPARs in adipocytes. In an in vivo study, a significant decrease in PPARα and PPARγ mRNA expression levels in adipose tissue was observed after 10 days of exposure to mercuric chloride (HgCl_2_) in high-fat diet-induced obesity in mice [41]. In this study, we observed positive associations between blood Hg concentration and the prevalence of hypercholesterolemia and hyper-LDL cholesterolemia in boys, but not in girls. The sex differences of Hg exposure on dyslipidemia could be attributed to the protective effect of estrogen. Previous studies have reported that estrogen acts as a scavenger of reactive oxygen species (ROS) [42]. Therefore, males may be more sensitive to oxidative stress than females, especially premenopausal females, which further results in an impaired lipid metabolism [43]. Furthermore, the sex differences may also link to PPARs. An in vivo study reported that male rats have higher levels of hepatic PPARα mRNA and protein than female rats [44]. Additionally, estrogen may have a reverse effect on PPARα target gene expression to maintain hepatic PPARα levels [45]. Further studies are needed to elucidate the mechanism of Hg on the development of dyslipidemia.

To our best knowledge, this study is the first study to identify the association between environmental Hg exposure and the prevalence of borderline dyslipidemia in adolescents. Moreover, the nationally representative data from the KNHANES give strength to the external validity of our results. However, there are several limitations to this study. First, findings from the cross-sectional study may be limited to interpret a causal relationship between blood Hg concentration and the risk of dyslipidemia in adolescents. Second, we could not adjust for daily Hg exposure from dental amalgam status [46]. Furthermore, dietary intake of fish and shellfish, another main routine of Hg exposure [47], may remain as a residual confounder, since a food frequency questionnaire (FFQ) was not carried out among adolescents in the KNHANES 2012–2013 and 2016. Third, we could not distinguish the specific species of Hg exposure, such as MeHg. However, a previous study has shown a similar effect of MeHg exposure on the risk of dyslipidemia [15]. Fourth, the subgroup analysis by BMI, physical activity, or other factors was not performed due to the small sample size. Hence, findings in this study need to be replicated in a larger study.

The findings in this study show that blood Hg concentration in adolescents are associated with borderline dyslipidemia. The GM of blood Hg concentration from 2010 to 2016 decreased among Korean adolescents (i.e., from 2.22 µg/L to 1.75 µg/L) [48,49,50,51,52]. However, blood Hg concentrations in this study (1.84 µg/L (median)) were still higher than those reported in other countries (e.g., US adolescents aged 12–19 years: 0.68 µg/L [arithmetic mean], Swedish adolescents aged 12, 15, and 18 years: 0.72 µg/L (median)) [53,54], although it was much lower than 5.00 µg/L, the reference level of whole-blood Hg concentrations for adolescents derived by the German Human Biomonitoring Commission [55]. Such a situation on Hg exposure does not only apply to Korea, but also to China (1.10 µg/L (GM)) and Japan (4.55 µg/L (GM)), etc. [56,57]. In other words, environmental Hg exposure may partly explain the increased prevalence of dyslipidemia among adolescents in East Asia. Recently, for a reduction of the risk of CVD, it has been emphasized that borderline dyslipidemia should be managed more strictly from early-childhood [58]. In this perspective, the current study suggests that Hg exposure should be more tightly controlled and the reference level of blood Hg concentrations should probably be lower than the previously recommended value [55].

## 5. Conclusions

In conclusion, blood Hg concentration is associated with the prevalence of borderline hypercholesterolemia among adolescents aged 10–18 years in Korea. This finding indicates that public health actions should be taken to reduce exposure to Hg for the prevention of dyslipidemia from early-childhood.

## Figures and Tables

**Table 1:** Baseline characteristics of the study participants according to TC and LDL-cholesterol levels.

	Overall (*n* = 1559)	TC				LDL-Cholesterol			
		Acceptable (*n* = 1034)	Borderline High (*n* = 395)	High (*n* = 130)	*p* ^a^	Acceptable (*n* = 1242)	Borderline High (*n* = 224)	High (*n* = 93)	*p* ^a^
Age, mean ± SE, years	14.2 ± 0.1	14.3 ± 0.1	13.8 ± 0.1	13.6 ± 0.3	<0.0001	14.2 ± 0.1	13.8 ± 0.2	14.1 ± 0.3	0.031
10–12 years, n (%)	524 (33.6)	316 (30.6)	152 (38.5)	56 (43.1)	0.003	402 (32.4)	87 (38.8)	35 (37.6)	0.208
13–15 years, n (%)	568 (36.4)	399 (38.6)	134 (33.9)	35 (26.9)		467 (37.6)	71 (31.7)	30 (32.3)	
16–18 years, n (%)	467 (30.0)	319 (30.9)	109 (27.6)	39 (30.0)		373 (30.0)	66 (29.5)	28 (30.1)	
Sex									
Boys, n (%)	806 (51.7)	582 (56.3)	171 (43.3)	53 (40.8)	<0.0001	658 (53.0)	109 (48.7)	39 (41.9)	0.006
Girls, n (%)	753 (48.3)	452 (43.7)	224 (56.7)	77 (59.2)		584 (47.0)	115 (51.3)	54 (58.1)	
BMI, mean ± SE, kg/m^2^	20.9 ± 0.1	20.7 ± 0.1	21.0 ± 0.2	21.7 ± 0.5	0.005	20.6 ± 0.1	21.6 ± 0.3	22.2 ± 0.6	<0.0001
Normal weight, n (%)	1229 (78.8)	843 (81.5)	297 (75.2)	89 (68.5)	0.005	1009 (81.2)	161 (71.9)	59 (63.4)	<0.0001
Overweight and obesity, n (%)	330 (21.2)	191 (18.5)	98 (24.8)	41 (31.5)		233 (18.8)	63 (28.1)	34 (36.6)	
Smoking status									
Never smokers, n (%)	1388 (89.0)	910 (88.0)	355 (89.9)	123 (94.6)	0.006	1098 (88.4)	204 (91.1)	86 (92.5)	0.138
Smokers, n (%)	171 (11.0)	124 (12.0)	40 (10.1)	7 (5.4)		144 (11.6)	20 (8.9)	7 (7.5)	
Household income level									
Quintile 1, n (%)	216 (13.9)	138 (13.3)	62 (15.7)	16 (12.3)	0.362	169 (13.6)	38 (17.0)	9 (9.7)	0.043
Quintile 2, n (%)	322 (20.7)	212 (20.5)	83 (21.0)	27 (20.8)		259 (20.9)	46 (20.5)	17 (18.3)	
Quintile 3, n (%)	343 (22.0)	223 (21.6)	94 (23.8)	26 (20.0)		267 (21.5)	58 (25.9)	18 (19.4)	
Quintile 4, n (%)	322 (20.7)	223 (21.6)	75 (19.0)	24 (18.5)		265 (21.3)	39 (17.4)	18 (19.4)	
Quintile 5, n (%)	356 (22.8)	238 (23.0)	81 (20.5)	37 (28.5)		282 (22.7)	43 (19.2)	31 (33.3)	
Physical activity									
Inactive, n (%)	703 (45.1)	421 (40.7)	219 (55.4)	63 (48.5)	0.001	547 (44.0)	113 (50.4)	43 (46.2)	0.180
Minimally active, n (%)	484 (31.0)	353 (34.1)	97 (24.6)	34 (26.2)		405 (32.6)	53 (23.7)	26 (28.0)	
Active, n (%)	372 (23.9)	260 (25.1)	79 (20.0)	33 (25.4)		290 (23.3)	58 (25.9)	24 (25.8)	
Menstruation (for girls)									
Premenarcheal, n (%)	169 (22.4)	90 (19.9)	62 (27.7)	17 (22.1)	0.012	129 (22.1)	27 (23.5)	13 (24.1)	0.540
Postmenarcheal, n (%)	584 (77.6)	362 (80.1)	162 (72.3)	60 (77.9)		455 (77.9)	88 (76.5)	41 (75.9)	
Total energy intake, mean ± SE, kcal/day	2188.9 ± 26.4	2267.5 ± 31.8	2036.3 ± 47.7	2016.1 ± 95.5	<0.0001	2237.1 ± 29.2	2006.6 ± 60.7	2032.2 ± 118.9	<0.0001
Survey year									
2010, n (%)	323 (20.7)	208 (20.1)	90 (22.8)	25 (19.2)	0.354	251 (20.2)	54 (24.1)	18 (19.4)	0.805
2011, n (%)	334 (21.4)	239 (23.1)	70 (17.7)	25 (19.2)		273 (22.0)	42 (18.8)	19 (20.4)	
2012, n (%)	318 (20.4)	217 (21.0)	71 (18.0)	30 (23.1)		259 (20.9)	38 (17.0)	21 (22.6)	
2013, n (%)	318 (20.4)	208 (20.1)	86 (21.8)	24 (18.5)		253 (20.4)	50 (22.3)	15 (16.1)	
2016, n (%)	266 (17.1)	162 (15.7)	78 (19.7)	26 (20.0)		206 (16.6)	40 (17.9)	20 (21.5)	
Blood Hg concentration, mean ± SE, µg/L	2.1 ± 0	2.1 ± 0	2.2 ± 0.1	2.2 ± 0.1	0.005	2.1 ± 0	2.2 ± 0.1	2.5 ± 0.2	0.002

Abbreviations: TC, total cholesterol; LDL, low-density lipoprotein; SE, standard error; BMI, body mass index; Hg, mercury. ^a^ *p*-value was calculated using ANOVA test for continuous variable and Rao-Scott Chi-Square test for categorical variable.

**Table 2:** Distribution of blood Hg concentration (µg/L) according to sex.

	*n* (%)	GM (95% CI)	Min	10%	25th	Median	75th	90%	Max
Overall	1559	1.88 (1.82, 1.94)	0.19	1.05	1.39	1.84	2.54	3.36	11.76
Boys	806 (51.7)	1.91 (1.83, 2.00)	0.19	1.03	1.40	1.87	2.59	3.48	11.76
Girls	753 (48.3)	1.84 (1.77, 1.91)	0.42	1.08	1.37	1.77	2.49	3.24	8.82

Abbreviations: GM, geometric mean; CI, confidence interval; Min, minimum; Max, Maximum.

**Table 3:** Baseline characteristics of the study participants according to sex.

	Boys (*n* = 806)	Girls (*n* = 753)	*p* ^a^
Age, mean ± SE, years	14.2 ± 0.1	14.1 ± 0.1	0.481
10–12 years, n (%)	277 (34.4)	247 (32.8)	0.896
13–15 years, n (%)	294 (36.5)	274 (36.4)	
16–18 years, n (%)	235 (29.2)	232 (30.8)	
BMI, mean ± SE, kg/m^2^	21.1 ± 0.2	20.6 ± 0.2	0.003
Normal weight, n (%)	629 (78.0)	600 (79.7)	0.677
Overweight and obesity, n (%)	177 (22.0)	153 (20.3)	
Smoking status			
Never smokers, n (%)	691 (85.7)	697 (92.6)	0.001
Smokers, n (%)	115 (14.3)	56 (7.4)	
Household income level			
Quintile 1, n (%)	109 (13.5)	107 (14.2)	0.484
Quintile 2, n (%)	160 (19.9)	162 (21.5)	
Quintile 3, n (%)	173 (21.5)	170 (22.6)	
Quintile 4, n (%)	179 (22.2)	143 (19.0)	
Quintile 5, n (%)	185 (23.0)	171 (22.7)	
Physical activity			
Inactive, n (%)	310 (38.5)	393 (52.2)	<0.0001
Minimally active, n (%)	229 (28.4)	255 (33.9)	
Active, n (%)	267 (33.1)	105 (13.9)	
Menstruation (for girls)			
Premenarcheal, n (%)	-	169 (22.4)	-
Postmenarcheal, n (%)	-	584 (77.6)	
Total energy intake, mean ± SE, kcal/day	2435.3 ± 39.6	1911.1 ± 30.5	<0.0001
Survey year			
2010, n (%)	164 (20.3)	159 (21.1)	0.895
2011, n (%)	172 (21.3)	162 (21.5)	
2012, n (%)	164 (20.3)	154 (20.5)	
2013, n (%)	160 (19.9)	158 (21.0)	
2016, n (%)	146 (18.1)	120 (15.9)	
Blood Hg concentration, mean ± SE, µg/L	2.2 ± 0.1	2.0 ± 0	0.110
TC, mean ± SE, mg/dL	154.4 ± 1.2	166.0 ± 1.3	<0.0001
Acceptable, n (%)	582 (72.2)	452 (60.0)	<0.0001
Borderline high, n (%)	171 (21.2)	224 (29.7)	
High, n (%)	53 (6.6)	77 (10.2)	
LDL-cholesterol, mean ± SE, mg/dL	88.5 ± 1.0	95.6 ± 1.2	<0.0001
Acceptable, n (%)	658 (81.6)	584 (77.6)	0.006
Borderline high, n (%)	109 (13.5)	115 (15.3)	
High, n (%)	39 (4.8)	54 (7.2)	
TG, mean ± SE, mg/dL	81.6 ± 2.0	86.3 ± 2.2	0.013
Acceptable, n (%)	546 (67.7)	479 (63.6)	0.734
Borderline high, n (%)	169 (21.0)	178 (23.6)	
High, n (%)	91 (11.3)	96 (12.7)	
HDL-cholesterol, mean ± SE, mg/dL	49.5 ± 0.4	53.1 ± 0.5	<0.0001
Acceptable, n (%)	550 (68.2)	583 (77.4)	0.002
Borderline low, n (%)	136 (16.9)	104 (13.8)	
Low, n (%)	120 (14.9)	66 (8.8)	

Abbreviations: SE, standard error; BMI, body mass index; Hg, mercury; TC, total cholesterol; LDL, low-density lipoprotein; TG, triglyceride; HDL, high-density lipoprotein. ^a^ *p*-value was calculated using independent *t*-test for continuous variable and Rao-Scott Chi-Square test for categorical variable.

**Table 4:** Associations between blood Hg concentration and prevalence of borderline hypercholesterolemia and hyper-LDL cholesterolemia according to sex.

	Concentration Range (µg/L)	Overall			Boys			Girls		
		Case/Total	OR (95% CI) ^a^	OR (95% CI) ^b^	Case/Total	OR (95% CI) ^a^	OR (95% CI) ^b^	Case/Total	OR (95% CI) ^a^	OR (95% CI) ^b^
Borderline hypercholesterolemia										
Continuous		395/1429	1.36 (1.02, 1.80)	1.48 (1.10, 2.00)	171/753	1.50 (1.03, 2.18)	1.63 (1.10, 2.41)	224/676	1.31 (0.85, 2.00)	1.23 (0.77, 1.96)
Tertile 1	0.192–1.531	120/482	1.00 (Reference)	1.00 (Reference)	42/240	1.00 (Reference)	1.00 (Reference)	78/242	1.00 (Reference)	1.00 (Reference)
Tertiles 2 and 3	1.532–11.761	275/947	1.27 (0.94, 1.72)	1.36 (0.99, 1.86)	129/513	1.70 (1.07, 2.71)	1.92 (1.19, 3.10)	146/434	1.04 (0.69, 1.58)	0.93 (0.59, 1.46)
Borderline hyper-LDL cholesterolemia										
Continuous		224/1466	1.28 (0.90, 1.83)	1.19 (0.81, 1.75)	109/767	1.33 (0.86, 2.05)	1.21 (0.74, 1.97)	115/699	1.28 (0.74, 2.22)	1.15 (0.62, 2.10)
Tertile 1	0.192–1.531	70/495	1.00 (Reference)	1.00 (Reference)	26/245	1.00 (Reference)	1.00 (Reference)	44/250	1.00 (Reference)	1.00 (Reference)
Tertiles 2 and 3	1.532–11.761	154/971	1.19 (0.79, 1.80)	1.10 (0.72, 1.69)	83/522	1.69 (0.95, 3.00)	1.63 (0.88, 3.04)	71/449	0.93 (0.55, 1.55)	0.79 (0.45, 1.38)

Abbreviations: LDL, low-density lipoprotein; OR, odds ratio; CI, confidence interval. ^a^ OR (95% CI) was calculated using univariate logistic regression. ^b^ OR (95% CI) was calculated using multivariate logistic regression adjusted for age (years, continuous), sex (for boys and girls combined), BMI (kg/m^2^, continuous), survey year (2010, 2011, 2012, 2013, and 2016), total energy intake (kcal/day, continuous), smoking status (never smokers and smokers), household income level (quintile 1, 2, 3, 4, and 5), physical activity (inactive, minimally active, and active), and menstruation (for girls, premenarcheal and postmenarcheal).

## Data Availability

The datasets generated and analyzed during the current study are available in the KNHANES repository, https://knhanes.kdca.go.kr/knhanes/eng/index.do.

## Supplementary Materials

The following are available online at https://www.mdpi.com/article/10.3390/toxics9100242/s1, Table S1: Classification of lipid and lipoprotein levels (mg/dL) in adolescents, Table S2: Baseline characteristics of the study participants according to TG and HDL-cholesterol levels, Table S3: Covariates according to blood Hg distribution, Table S4: Associations between blood Hg concentration and prevalence of hypercholesterolemia and hyper-LDL cholesterolemia according to sex, Table S5: Associations between blood Hg concentration and prevalence of overt hypercholesterolemia and hyper-LDL cholesterolemia according to sex.

## References

[1] Fernandes Azevedo B., Barros Furieri L., Peçanha F.M., Wiggers G.A., Frizera Vassallo P., Ronacher Simões M., Fiorim J., Rossi de Batista P., Fioresi M., Rossoni L. (2012). Toxic Effects of Mercury on the Cardiovascular and Central Nervous Systems. J. Biomed. Biotechnol..

[2] Virtanen J.K., Rissanen T.H., Voutilainen S., Tuomainen T.-P. (2007). Mercury as a risk factor for cardiovascular diseases. J. Nutr. Biochem..

[3] Hodgson S., Nieuwenhuijsen M.J., Elliott P., Jarup L. (2007). Kidney Disease Mortality and Environmental Exposure to Mercury. Am. J. Epidemiol..

[4] Roy C., Tremblay P.-Y., Ayotte P. (2017). Is mercury exposure causing diabetes, metabolic syndrome and insulin resistance? A systematic review of the literature. Environ. Res..

[5] Virtanen J.K., Voutilainen S., Rissanen T.H., Mursu J., Tuomainen T.-P., Korhonen M.J., Valkonen V.-P., Seppänen K., Laukkanen J.A., Salonen J.T. (2005). Mercury, Fish Oils, and Risk of Acute Coronary Events and Cardiovascular Disease, Coronary Heart Disease, and All-Cause Mortality in Men in Eastern Finland. Arterioscler. Thromb. Vasc. Biol..

[6] Hu X.F., Lowe M., Chan H.M. (2021). Mercury exposure, cardiovascular disease, and mortality: A systematic review and dose-response meta-analysis. Environ. Res..

[7] World Health Organization (2017). Cardiovascular Diseases (CVDs).

[8] NCD Risk Factor Collaboration (2020). National trends in total cholesterol obscure heterogeneous changes in HDL and non-HDL cholesterol and total-to-HDL cholesterol ratio: A pooled analysis of 458 population-based studies in Asian and Western countries. Int. J. Epidemiol..

[9] Pirillo A., Casula M., Olmastroni E., Norata G.D., Catapano A.L. (2021). Global epidemiology of dyslipidaemias. Nat. Rev. Cardiol..

[10] Korea Centers for Disease Control and Prevention (2012). Korea Health Statistics 2005: Korea National Health and Nutrition Examination Survey (KNHANES III).

[11] Korea Centers for Disease Control and Prevention (2020). Korea Health Statistics 2019: Korea National Health and Nutrition Examination Survey (KNHANES VIII-1).

[12] Berenson G.S., Srinivasan S.R., Bao W., Newman W.P., Tracy R.E., Wattigney W.A. (1998). Association between Multiple Cardiovascular Risk Factors and Atherosclerosis in Children and Young Adults. N. Engl. J. Med..

[13] McGill H.C., McMahan C.A., Zieske A.W., Sloop G.D., Walcott J.V., Troxclair D.A., Malcom G.T., Tracy R.E., Oalmann M.C., Strong J.P. (2000). Associations of Coronary Heart Disease Risk Factors With the Intermediate Lesion of Atherosclerosis in Youth. Arterioscler. Thromb. Vasc. Biol..

[14] Fan Y., Zhang C., Bu J. (2017). Relationship between Selected Serum Metallic Elements and Obesity in Children and Adolescent in the U.S. Nutrients.

[15] Zhang Y., Xu C., Fu Z., Shu Y., Zhang J., Lu C., Mo X. (2018). Associations between total mercury and methyl mercury exposure and cardiovascular risk factors in US adolescents. Environ. Sci. Pollut. Res..

[16] Huang Y., Cai X., Liu C., Zhu D., Hua J., Hu Y., Peng J., Xu D. (2015). Prehypertension and the Risk of Coronary Heart Disease in Asian and Western Populations: A Meta-analysis. J. Am. Heart Assoc..

[17] Expert Panel on Integrated Guideline for Cardiovascular Health and Risk Reduction in Children and Adolescents, National Heart, Lung and Blood Institute (2011). Expert panel on integrated guidelines for cardiovascular health and risk reduction in children and adolescents: Summary report. Pediatrics.

[18] Kweon S., Kim Y., Jang M.-j., Kim Y., Kim K., Choi S., Chun C., Khang Y.-H., Oh K. (2014). Data Resource Profile: The Korea National Health and Nutrition Examination Survey (KNHANES). Int. J. Epidemiol..

[19] Kim N.-S., Lee B.-K. (2011). National estimates of blood lead, cadmium, and mercury levels in the Korean general adult population. Int. Arch. Occup. Environ. Health.

[20] Friedewald W.T., Levy R.I., Fredrickson D.S. (1972). Estimation of the Concentration of Low-Density Lipoprotein Cholesterol in Plasma, Without Use of the Preparative Ultracentrifuge. Clin. Chem..

[21] Craig C.L., Marshall A.L., Sjöström M., Bauman A.E., Booth M.L., Ainsworth B.E., Pratt M., Ekelund U., Yngve A., Sallis J.F. (2003). International physical activity questionnaire: 12-country reliability and validity. Med. Sci. Sports Exerc..

[22] Herrmann S.D., Heumann K.J., Der Ananian C.A., Ainsworth B.E. (2013). Validity and Reliability of the Global Physical Activity Questionnaire (GPAQ). Meas. Phys. Educ. Exerc. Sci..

[23] Ministry of Education, Ministry of Health and Welfare, Korea Centers for Disease Control and Prevention (2020). The 16th Korea Youth Risk Behavior Web-Based Survey.

[24] Park S. (2014). Associations of physical activity with sleep satisfaction, perceived stress, and problematic Internet use in Korean adolescents. BMC Public Health.

[25] Rao J.N.K., Scott A.J. (1984). On Chi-Squared Tests for Multiway Contingency Tables with Cell Proportions Estimated from Survey Data. Ann. Stat..

[26] SAS Institute Inc. (2011). SAS/STAT® 9.3 User’s Guide.

[27] SAS Institute Inc. (2016). SAS/STAT® 14.2 User’s Guide.

[28] Song K., Jeon S., Lee H.S., Choi H.S., Suh J., Kwon A., Kim H.-S., Chae H.W. (2021). Trends of Dyslipidemia in Korean Youth According to Sex and Body Mass Index: Based on the Korea National Health and Nutrition Examination Survey (2007–2018). J. Pediatrics.

[29] Ding W., Cheng H., Yan Y., Zhao X., Chen F., Huang G., Hou D., Mi J. (2016). 10-Year Trends in Serum Lipid Levels and Dyslipidemia Among Children and Adolescents From Several Schools in Beijing, China. J. Epidemiol..

[30] Kouda K., Iki M., Fujita Y., Nakamura H., Ohara K., Tachiki T., Nishiyama T. (2020). Trends in Serum Lipid Levels of a 10- and 13-Year-Old Population in Fukuroi City, Japan (2007–2017). J. Epidemiol..

[31] Cho H.W., Kim S.-H., Park M.J. (2020). An association of blood mercury levels and hypercholesterolemia among Korean adolescents. Sci. Total Environ..

[32] Madamanchi N.R., Vendrov A., Runge M.S. (2005). Oxidative Stress and Vascular Disease. Arterioscler. Thromb. Vasc. Biol..

[33] Mackness M., Mackness B. (2004). Paraoxonase 1 and atherosclerosis: Is the gene or the protein more important?. Free Radic. Biol. Med..

[34] Bhattacharyya T., Nicholls S.J., Topol E.J., Zhang R., Yang X., Schmitt D., Fu X., Shao M., Brennan D.M., Ellis S.G. (2008). Relationship of Paraoxonase 1 (PON1) Gene Polymorphisms and Functional Activity With Systemic Oxidative Stress and Cardiovascular Risk. JAMA.

[35] Mackness M.I., Mackness B., Durrington P.N., Fogelman A.M., Berliner J., Lusis A.J., Navab M., Shih D., Fonarow G.C. (1998). Paraoxonase and coronary heart disease. Curr. Opin. Lipidol..

[36] Tinkov A.A., Ajsuvakova O.P., Skalnaya M.G., Popova E.V., Sinitskii A.I., Nemereshina O.N., Gatiatulina E.R., Nikonorov A.A., Skalny A.V. (2015). Mercury and metabolic syndrome: A review of experimental and clinical observations. Biometals.

[37] Ayotte P., Carrier A., Ouellet N., Boiteau V., Abdous B., Sidi Elhadji Anassour L., Château-Degat M.-L., Dewailly É. (2011). Relation between Methylmercury Exposure and Plasma Paraoxonase Activity in Inuit Adults from Nunavik. Environ. Health Perspect..

[38] Aviram M. (2000). Review of human studies on oxidative damage and antioxidant protection related to cardiovascular diseases. Free Radic. Res..

[39] Chinetti G., Fruchart J.C., Staels B. (2000). Peroxisome proliferator-activated receptors (PPARs): Nuclear receptors at the crossroads between lipid metabolism and inflammation. Inflamm. Res..

[40] De Freitas Bonomo L., Silva M., de Paula Oliveira R., Silva M.E., Pedrosa M.L. (2012). Iron overload potentiates diet-induced hypercholesterolemia and reduces liver ppar-α expression in hamsters. J. Biochem. Mol. Toxicol..

[41] Kawakami T., Hanao N., Nishiyama K., Kadota Y., Inoue M., Sato M., Suzuki S. (2012). Differential effects of cobalt and mercury on lipid metabolism in the white adipose tissue of high-fat diet-induced obesity mice. Toxicol. Appl. Pharmacol..

[42] Olivieri G., Novakovic M., Savaskan E., Meier F., Baysang G., Brockhaus M., Müller-Spahn F. (2002). The effects of β-estradiol on SHSY5Y neuroblastoma cells during heavy metal induced oxidative stress, neurotoxicity and β-amyloid secretion. Neuroscience.

[43] Benz V., Kintscher U., Foryst-Ludwig A., Regitz-Zagrosek V. (2012). Sex-Specific Differences in Type 2 Diabetes Mellitus and Dyslipidemia Therapy: PPAR Agonists. Sex and Gender Differences in Pharmacology.

[44] Jalouli M., Carlsson L., Améen C., Lindén D., Ljungberg A., Michalik L., Edén S., Wahli W., Oscarsson J. (2003). Sex Difference in Hepatic Peroxisome Proliferator-Activated Receptor α Expression: Influence of Pituitary and Gonadal Hormones. Endocrinology.

[45] Jeong S., Yoon M. (2007). Inhibition of the Actions of Peroxisome Proliferator-activated Receptor α on Obesity by Estrogen. Obesity.

[46] Siblerud R.L. (1990). The relationship between mercury from dental amalgam and the cardiovascular system. Sci. Total Environ..

[47] Björkman L., Lundekvam B.F., Lægreid T., Bertelsen B.I., Morild I., Lilleng P., Lind B., Palm B., Vahter M. (2007). Mercury in human brain, blood, muscle and toenails in relation to exposure: An autopsy study. Environ. Health.

[48] Korea Centers for Disease Control and Prevention (2012). Korea Health Statistics 2010: Korea National Health and Nutrition Examination Survey (KNHANES V-1).

[49] Korea Centers for Disease Control and Prevention (2012). Korea Health Statistics 2011: Korea National Health and Nutrition Examination Survey (KNHANES V-2).

[50] Korea Centers for Disease Control and Prevention (2013). Korea Health Statistics 2012: Korea National Health and Nutrition Examination Survey (KNHANES V-3).

[51] Korea Centers for Disease Control and Prevention (2014). Korea Health Statistics 2013: Korea National Health and Nutrition Examination Survey (KNHANES VI-1).

[52] Korea Centers for Disease Control and Prevention (2017). Korea Health Statistics 2016: Korea National Health and Nutrition Examination Survey (KNHANES VII-1).

[53] Sanders A.P., Mazzella M.J., Malin A.J., Hair G.M., Busgang S.A., Saland J.M., Curtin P. (2019). Combined exposure to lead, cadmium, mercury, and arsenic and kidney health in adolescents age 12–19 in NHANES 2009–2014. Environ. Int..

[54] Almerud P., Zamaratskaia G., Lindroos A.K., Bjermo H., Andersson E.M., Lundh T., Ankarberg E.H., Lignell S. (2021). Cadmium, total mercury, and lead in blood and associations with diet, sociodemographic factors, and smoking in Swedish adolescents. Environ. Res..

[55] Apel P., Angerer J., Wilhelm M., Kolossa-Gehring M. (2017). New HBM values for emerging substances, inventory of reference and HBM values in force, and working principles of the German Human Biomonitoring Commission. Int. J. Hyg. Environ. Health.

[56] Gao Z.-Y., Li M.-M., Wang J., Yan J., Zhou C.-C., Yan C.-H. (2018). Blood mercury concentration, fish consumption and anthropometry in Chinese children: A national study. Environ. Int..

[57] Ilmiawati C., Yoshida T., Itoh T., Nakagi Y., Saijo Y., Sugioka Y., Sakamoto M., Ikegami A., Ogawa M., Kayama F. (2015). Biomonitoring of mercury, cadmium, and lead exposure in Japanese children: A cross-sectional study. Environ. Health Prev. Med..

[58] Grundy Scott M., Stone Neil J., Bailey Alison L., Beam C., Birtcher Kim K., Blumenthal Roger S., Braun Lynne T., de Ferranti S., Faiella-Tommasino J., Forman Daniel E. (2019). 2018 AHA/ACC/AACVPR/AAPA/ABC/ACPM/ADA/AGS/APhA/ASPC/NLA/PCNA Guideline on the Management of Blood Cholesterol. J. Am. Coll. Cardiol..

