# Supplementary material for: Association between Blood Mercury Concentration and Prevalence of Borderline Hypercholesterolemia among Adolescents: The Korea National Health and Nutrition Examination Survey (KNHANES) 2010–2013 and 2016
Source: Toxics. 2021 Sep 29;9(10):242. doi: 10.3390/toxics9100242 (PMC8539976; doi:10.3390/toxics9100242)
Supplement: Supplementary file 1 [file toxics-09-00242-s001.zip › toxics-1391388-supplementary.pdf]

Article

Supplementary Materials: Association Between Blood Mercury Concentration and Prevalence of Borderline Hypercholesterolemia among Adolescents: the Korea National Health and Nutrition Examination Survey (KNHANES) 2010-2013 and 2016

Taiyue Jin, Eun Young Park, Byungmi Kim and Jin-Kyoung Oh

Table S1. Classification of lipid and lipoprotein levels (mg/dL) in adolescents.

		Acceptable	Borderline	Overt
Dyslipidemia [a]				
Hypercholesterolemia	TC	< 170	170-199	≥ 200
Hypertriglyceridemia	TG	< 90	90-129	≥ 130
Hyper-LDL cholesterolemia	LDL-cholesterol	< 110	110-129	≥ 130
Hypo-HDL cholesterolemia	HDL-cholesterol	> 45	40-45	< 40

Abbreviations: TC, total cholesterol; TG, triglyceride; LDL, low-density lipoprotein; HDL, high-density lipoprotein. [a] Dyslipidemia was defined as any one or more of hypercholesterolemia, hyper-LDL cholesterolemia, hypertriglyceridemia, or hypo-HDL cholesterolemia.

Table S2. Baseline characteristics of the study participants according to TG and HDL-cholesterol levels.

	TG				HDL-Cholesterol			
	Acceptable (*n* = 1,025)	Borderline high (*n* = 347)	High (*n* = 187)	*p* [a]	Acceptable (*n* = 1,133)	Borderline low (*n* = 240)	Low (*n* = 186)	*p* [a]
Age, mean ± SE, years	14.2 ± 0.1	14.2 ± 0.2	14.0 ± 0.2	0.421	14.0 ± 0.1	14.4 ± 0.2	14.5 ± 0.2	0.002
10-12 years, n (%)	336 (32.8)	122 (35.2)	66 (35.3)	0.153	405 (35.7)	70 (29.2)	49 (26.3)	0.008
13-15 years, n (%)	387 (37.8)	114 (32.9)	67 (35.8)		395 (34.9)	91 (37.9)	82 (44.1)	
16-18 years, n (%)	302 (29.5)	111 (32.0)	54 (28.9)		333 (29.4)	79 (32.9)	55 (29.6)	
Sex								
Boys, n (%)	546 (53.3)	169 (48.7)	91 (48.7)	0.734	550 (48.5)	136 (56.7)	120 (64.5)	0.002
Girls, n (%)	479 (46.7)	178 (51.3)	96 (51.3)		583 (51.5)	104 (43.3)	66 (35.5)	
BMI, mean ± SE, kg/m^2	20.2 ± 0.1	21.5 ± 0.2	23.1 ± 0.4	<0.0001	20.3 ± 0.1	22.1 ± 0.3	22.4 ± 0.3	<0.0001
Normal weight, n (%)	867 (84.6)	254 (73.2)	108 (57.8)	<0.0001	944 (83.3)	171 (71.3)	114 (61.3)	<0.0001
Overweight and obesity, n (%)	158 (15.4)	93 (26.8)	79 (42.2)		189 (16.7)	69 (28.8)	72 (38.7)	
Smoking status								
Never smokers, n (%)	910 (88.8)	306 (88.2)	172 (92.0)	0.224	1,017 (89.8)	208 (86.7)	163 (87.6)	0.424
Smokers, n (%)	115 (11.2)	41 (11.8)	15 (8.0)		116 (10.2)	32 (13.3)	23 (12.4)	
Household income level								
Quintile 1, n (%)	127 (12.4)	58 (16.7)	31 (16.6)	0.595	154 (13.6)	34 (14.2)	28 (15.1)	0.851
Quintile 2, n (%)	200 (19.5)	76 (21.9)	46 (24.6)		228 (20.1)	53 (22.1)	41 (22.0)	
Quintile 3, n (%)	235 (22.9)	66 (19.0)	42 (22.5)		256 (22.6)	52 (21.7)	35 (18.8)	
Quintile 4, n (%)	214 (20.9)	77 (22.2)	31 (16.6)		232 (20.5)	45 (18.8)	45 (24.2)	
Quintile 5, n (%)	249 (24.3)	70 (20.2)	37 (19.8)		263 (23.2)	56 (23.3)	37 (19.9)	
Physical activity								
Inactive, n (%)	461 (45.0)	158 (45.5)	84 (44.9)	0.435	538 (47.5)	95 (39.6)	70 (37.6)	0.002
Minimally active, n (%)	308 (30.0)	114 (32.9)	62 (33.2)		327 (28.9)	93 (38.8)	64 (34.4)	
Active, n (%)	256 (25.0)	75 (21.6)	41 (21.9)		268 (23.7)	52 (21.7)	52 (28.0)	
Menstruation (for girls)								
Premenarcheal, n (%)	102 (21.3)	39 (21.9)	28 (29.2)	0.345	130 (22.3)	24 (23.1)	15 (22.7)	0.623

Postmenarcheal, n (%)	377 (78.7)	139 (78.1)	68 (70.8)		453 (77.7)	80 (76.9)	51 (77.3)	
Total energy intake, mean ± SE, kcal/day	2,209.9 ± 31.3	2,153.6 ± 58.0	2,141.6 ± 80.2	0.126	2,171.0 ± 30.7	2,204.6 ± 75.6	2,273.5 ± 83.9	0.099
Survey year								
2010, n (%)	193 (18.8)	83 (23.9)	47 (25.1)	0.332	215 (19.0)	57 (23.8)	51 (27.4)	0.166
2011, n (%)	236 (23.0)	57 (16.4)	41 (21.9)		245 (21.6)	51 (21.3)	38 (20.4)	
2012, n (%)	218 (21.3)	65 (18.7)	35 (18.7)		241 (21.3)	49 (20.4)	28 (15.1)	
2013, n (%)	202 (19.7)	84 (24.2)	32 (17.1)		224 (19.8)	55 (22.9)	39 (21.0)	
2016, n (%)	176 (17.2)	58 (16.7)	32 (17.1)		208 (18.4)	28 (11.7)	30 (16.1)	
Blood Hg concentration, mean ± SE, μg/L	2.1 ± 0	2.1 ± 0.1	2.0 ± 0.1	0.841	2.1 ± 0	2.0 ± 0.1	2.2 ± 0.1	0.687

Abbreviations: HDL, high-density lipoprotein; SE, standard error; BMI, body mass index; Hg, mercury. [a] *p*-value was calculated using ANOVA test for continuous variable and Rao-Scott Chi-Square test for categorical variable.

Table S3. Covariates according to blood Hg distribution.

	Blood Hg concentration			
	**Tertile 1 (*n* = 519)**	**Tertile 2 (*n* = 520)**	**Tertile 3 (*n* = 520)**	***p*** [a]
Age, mean ± SE, years	14.1 ± 0.1	14.2 ± 0.1	14.2 ± 0.1	0.745
10-12 years, n (%)	170 (32.8)	180 (34.6)	174 (33.5)	0.263
13-15 years, n (%)	184 (35.5)	177 (34.0)	207 (39.8)	
16-18 years, n (%)	165 (31.8)	163 (31.3)	139 (26.7)	
Sex				
Boys, n (%)	251 (48.4)	270 (51.9)	285 (54.8)	0.188
Girls, n (%)	268 (51.6)	250 (48.1)	235 (45.2)	
BMI, mean ± SE, kg/m^2	20.5 ± 0.2	20.8 ± 0.2	21.3 ± 0.2	0.0004
Normal weight, n (%)	436 (84.0)	414 (79.6)	379 (72.9)	0.022
Overweight and obesity, n (%)	83 (16.0)	106 (20.4)	141 (27.1)	
Smoking status				
Never smokers, n (%)	472 (90.9)	454 (87.3)	462 (88.8)	0.396
Smokers, n (%)	47 (9.1)	66 (12.7)	58 (11.2)	
Household income level				
Quintile 1, n (%)	77 (14.8)	63 (12.1)	76 (14.6)	0.034
Quintile 2, n (%)	108 (20.8)	122 (23.5)	92 (17.7)	
Quintile 3, n (%)	133 (25.6)	109 (21.0)	101 (19.4)	
Quintile 4, n (%)	106 (20.4)	97 (18.7)	119 (22.9)	
Quintile 5, n (%)	95 (18.3)	129 (24.8)	132 (25.4)	
Physical activity				
Inactive, n (%)	252 (48.6)	227 (43.7)	224 (43.1)	0.102
Minimally active, n (%)	164 (31.6)	161 (31.0)	159 (30.6)	
Active, n (%)	103 (19.8)	132 (25.4)	137 (26.3)	
Menstruation (for girls)				
Premenarcheal, n (%)	46 (17.2)	65 (26.0)	58 (24.7)	0.010
Postmenarcheal, n (%)	222 (82.8)	185 (74.0)	177 (75.3)	
Total energy intake, mean ± SE, kcal/day	2090.2 ± 39.9	2170.5 ± 46.1	2300.6 ± 53.0	0.0002
Survey year				
2010, n (%)	62 (11.9)	107 (20.6)	154 (29.6)	<0.0001
2011, n (%)	108 (20.8)	109 (21.0)	117 (22.5)	
2012, n (%)	106 (20.4)	125 (24.0)	87 (16.7)	
2013, n (%)	133 (25.6)	99 (19.0)	86 (16.5)	
2016, n (%)	110 (21.2)	80 (15.4)	76 (14.6)	

Abbreviations: Hg, mercury; SE, standard error; BMI, body mass index. [a] *p*-value was calculated using ANOVA test for continuous variable and Rao-Scott Chi-Square test for categorical variable.

Table S4. Associations between blood Hg concentration and prevalence of hypercholesterolemia and hyper-LDL cholesterolemia according to sex.

	Concentration range (μg/L)	Overall			Boys			Girls		
		Case/total	OR (95% CI) [a]	OR (95% CI) [b]	Case/total	OR (95% CI) [a]	OR (95% CI) [b]	Case/total	OR (95% CI) [a]	OR (95% CI) [b]
Hypercholesterolemia										
Continuous		525/1,559	1.39 (1.07, 1.80)	1.50 (1.15, 1.97)	224/806	1.63 (1.17, 2.28)	1.74 (1.23, 2.46)	301/753	1.28 (0.86, 1.89)	1.23 (0.80, 1.90)
Tertile 1	0.192–1.531	157/519	1.00 (Reference)	1.00 (Reference)	53/251	1.00 (Reference)	1.00 (Reference)	104/268	1.00 (Reference)	1.00 (Reference)
Tertiles 2 and 3	1.532–11.761	368/1,040	1.32 (0.99, 1.76)	1.41 (1.05, 1.90)	171/555	1.82 (1.19, 2.79)	2.02 (1.30, 3.14)	197/485	1.08 (0.73, 1.59)	1.01 (0.66, 1.54)
Hyper-LDL cholesterolemia										
Continuous		317/1,559	1.42 (1.05, 1.94)	1.37 (0.99, 1.90)	148/806	1.78 (1.20, 2.64)	1.65 (1.06, 2.57)	169/753	1.20 (0.75, 1.91)	1.13 (0.68, 1.87)
Tertile 1	0.192–1.531	94/519	1.00 (Reference)	1.00 (Reference)	32/251	1.00 (Reference)	1.00 (Reference)	62/268	1.00 (Reference)	1.00 (Reference)
Tertiles 2 and 3	1.532–11.761	223/1,040	1.23 (0.86, 1.76)	1.18 (0.82, 1.70)	116/555	1.94 (1.16, 3.24)	1.88 (1.10, 3.20)	107/485	0.91 (0.58, 1.43)	0.83 (0.51, 1.35)

Abbreviations: LDL, low-density lipoprotein; OR, odds ratio; CI, confidence interval. [a] OR (95% CI) was calculated using univariate logistic regression. [b] OR (95% CI) was calculated using multivariate logistic regression adjusted for age (years, continuous), sex (for boys and girls combined), BMI (kg/m^2, continuous), survey year (2010, 2011, 2012, 2013, and 2016), total energy intake (kcal/day, continuous), smoking status (never smokers and smokers), household income level (quintile 1, 2, 3, 4, and 5), physical activity (inactive, minimally active, and active), and menstruation (for girls, premenarcheal and postmenarcheal).

Table S5. Associations between blood Hg concentration and prevalence of overt hypercholesterolemia and hyper-LDL cholesterolemia according to sex.

	Concentration range (μg/L)	Overall			Boys			Girls		
		Case/total	OR (95% CI) [a]	OR (95% CI) [b]	Case/total	OR (95% CI) [a]	OR (95% CI) [b]	Case/total	OR (95% CI) [a]	OR (95% CI) [b]
Overt hypercholesterolemia										
Continuous		130/1,164	1.47 (0.96, 2.25)	1.61 (1.03, 2.53)	53/635	2.13 (1.24, 3.66)	2.04 (1.15, 3.64)	77/529	1.20 (0.63, 2.30)	1.29 (0.64, 2.61)
Tertile 1	0.192–1.531	37/399	1.00 (Reference)	1.00 (Reference)	11/209	1.00 (Reference)	1.00 (Reference)	26/190	1.00 (Reference)	1.00 (Reference)
Tertiles 2 and 3	1.532–9.618	93/765	1.49 (0.89, 2.48)	1.51 (0.90, 2.53)	42/426	2.30 (1.02, 5.19)	2.36 (1.00, 5.54)	51/339	1.19 (0.62, 2.29)	1.22 (0.63, 2.35)
Overt hyper-LDL cholesterolemia										
Continuous		93/1,335	1.85 (1.07, 3.21)	1.79 (1.04, 3.08)	39/697	3.92 (2.10, 7.35)	3.20 (1.55, 6.62)	54/638	1.03 (0.44, 2.42)	1.10 (0.47, 2.55)
Tertile 1	0.192–1.531	24/449	1.00 (Reference)	1.00 (Reference)	6/225	1.00 (Reference)	1.00 (Reference)	18/224	1.00 (Reference)	1.00 (Reference)
Tertiles 2 and 3	1.532–11.761	69/886	1.34 (0.74, 2.45)	1.29 (0.71, 2.33)	33/472	3.09 (1.10, 8.73)	2.64 (0.94, 7.36)	36/414	0.88 (0.41, 1.89)	0.91 (0.43, 1.94)

Abbreviations: LDL, low-density lipoprotein; OR, odds ratio; CI, confidence interval. [a] OR (95% CI) was calculated using univariate logistic regression. [b] OR (95% CI) was calculated using multivariate logistic regression adjusted for age (years, continuous), sex (for boys and girls combined), BMI (kg/m^2, continuous), survey year (2010, 2011, 2012, 2013, and 2016), total energy intake (kcal/day, continuous), smoking status (never smokers and smokers), household income level (quintile 1, 2, 3, 4, and 5), physical activity (inactive, minimally active, and active), and menstruation (for girls, premenarcheal and postmenarcheal).
